# Multiorgan repair by MSC-derived extracellular vesicles in hepatorenal syndrome through necroptosis alleviation, immune reprogramming and fibrosis resolution

**DOI:** 10.20517/evcna.2025.99

**Published:** 2026-01-12

**Authors:** Kai-Chao Zhang, Shi-Han Mu, Run-Fang Song, Yu-Ru Gao, Sha Zhang, Chen-Xi Zheng, Yan Jin, Zhen Gong, Bing-Dong Sui, Min Zhang

**Affiliations:** ^1^College of Life Science, Northwest University, Xi’an 710069, Shaanxi, China.; ^2^State Key Laboratory of Military Stomatology & National Clinical Research Center for Oral Disease, Department of General Dentistry and Emergency, School of Stomatology, Fourth Military Medical University, Xi’an 710032, Shaanxi, China.; ^3^State Key Laboratory of Oral & Maxillofacial Reconstruction and Regeneration, National Clinical Research Center for Oral Diseases, Shaanxi International Joint Research Center for Oral Diseases, Center for Tissue Engineering, School of Stomatology, Fourth Military Medical University, Xi’an 710032, Shaanxi, China.; ^4^Department of Orthodontics, School of Stomatology, The Fourth Military Medical University, Xi’an 710032, Shaanxi, China.; ^5^Department of Traditional Chinese Medicine, The First Affiliated Hospital of Fourth Military Medical University, Xi’an 710032, Shaanxi, China.; ^6^College of Basic Medicine, Shaanxi Key Laboratory of Research on TCM Physical Constitution and Diseases Prevention and Treatment, Shaanxi University of Chinese Medicine, Xianyang 712046, Shaanxi, China.; ^7^Analysis & Testing Laboratory for Life Sciences and Medicine, Fourth Military Medical University, Xi’an 710032, Shaanxi, China.

**Keywords:** Mesenchymal stem cell, extracellular vesicles, hepatorenal syndrome, necroptosis, immune modulation, fibrosis

## Abstract

**Aim:** To investigate the therapeutic potential and underlying mechanism of mesenchymal stem cell (MSC)-derived extracellular vesicles (MSC-EVs) in treating hepatorenal syndrome (HRS), a condition lacking therapies for multi-organ damage.

**Methods:** EVs were isolated from human umbilical cord MSCs and characterized by transmission electron microscopy, nanoparticle tracking analysis, and proteomics. A murine model of HRS, induced by bile duct ligation (BDL), was established, and mice received intravenous MSC-EVs treatment. Therapeutic efficacy was assessed through histopathology, serum biochemistry, and analysis of necroptosis, inflammation, and fibrosis markers.

**Results:** Proteomic profiling of MSC-EVs revealed significant enrichment of proteins involved in renal processes, anti-fibrosis, and immune regulation. In BDL-induced HRS mice, MSC-EVs treatment demonstrated potent multi-organ protective effects. This was evidenced by alleviation of hepatic necroptosis and renal tubular injury, downregulation of interleukin-17 expression, and concurrent attenuation of fibrosis in both liver and kidney tissues. Consequently, significant improvements in hepatic and renal function markers were observed.

**Conclusion:** MSC-EVs represent a novel and effective cell-free nanotherapeutic strategy for HRS. They confer protection through multi-faceted mechanisms, including inhibition of necroptosis, immune reprogramming, and fibrosis resolution, offering a promising paradigm for the treatment of multi-organ failure.

## INTRODUCTION

Renal dysfunction often accompanies the progression of liver disease. In this context, multiple pathological factors drive the onset of hepatorenal syndrome (HRS), which imposes a substantial burden on patients due to its high morbidity and mortality^[1-3]^. Current evidence indicates that renal impairment in liver disease primarily stems from functional alterations in renal circulation during hepatic injury that exceed physiological compensatory capacity, ultimately compromising glomerular filtration rate. Systemic inflammation and bacterial translocation may further accelerate this disease progression^[1-3]^. Clinically, the mainstay of treatment combines vasoconstrictors with albumin, while liver transplantation or combined liver-kidney transplantation represents the optimal therapeutic approach^[4-6]^. However, the prognosis of HRS remains poor. Therefore, developing effective pharmacological interventions for HRS is an urgent unmet need.

Systemic inflammation and immune dysregulation are recognized as critical pathogenic drivers in HRS. Patients with HRS demonstrate significantly elevated serum levels of pro-inflammatory cytokines compared to cirrhotic patients without acute kidney injury, including interleukin (IL)-6, IL-17, and C-reactive protein (CRP)^[7-11]^. Notably, pathogen-associated molecular patterns (PAMPs) and damage-associated molecular patterns (DAMPs) are key instigators of systemic inflammation in HRS. PAMPs originate from the gut microbiota, whereas DAMPs are released by injured hepatocytes. These molecules collectively activate innate immunity, triggering the release of inflammatory mediators. In response, systemic arterial vasodilation intensifies, impairing circulatory homeostasis and exacerbating renal hypoperfusion. Beyond systemic effects, PAMPs and DAMPs may also directly promote renal tubular injury^[12-14]^. Nevertheless, effective strategies to alleviate inflammation in HRS remain lacking.

Mesenchymal stem cells (MSCs) represent a promising therapeutic strategy for inflammatory disorders due to their robust capacity to modulate immune cell phenotypes and function^[15-17]^. Recent advances have revealed that the immunomodulatory effects of MSCs are primarily mediated by secreted soluble factors and extracellular vesicles (EVs)^[18-21]^. EVs, nanoscale membrane-bound particles, serve as natural carriers of bioactive molecules - e.g., nucleic acids, proteins, lipids - that facilitate intercellular communication. Accumulating evidence indicates that MSC-derived EVs (MSC-EVs) exhibit therapeutic efficacy in models of liver fibrosis^[22-26]^ and renal injury^[27-30]^ by targeting multiple cell types, including immune cells^[31-35]^. Critically, as a cell-free alternative, MSC-EVs circumvent key limitations of whole-cell MSC therapy, such as tumorigenic risks, pulmonary entrapment, and inconsistent engraftment. Notably, whether MSC-EVs can be used for HRS treatment has not been reported.

This study tests the hypothesis that MSC-EVs may mitigate HRS through immunomodulation. After nanoscale characterization and proteomic profiling of EVs from umbilical cord MSCs, we identified MSC-EV cargo proteins implicated in fibrosis resolution and renal protection. In a bile duct ligation (BDL)-induced mouse model, intravenous administration of MSC-EVs attenuated multiorgan injury, as evidenced by reduced hepatic and renal fibrosis, ameliorated tissue damage and cell necroptosis, and functional restoration. Mechanistically, MSC-EVs were enriched with immune-regulatory proteins and correlated with suppressed IL-17 expression in target organs, as well as reduced serum IL-17 levels, indicating microenvironment reprogramming. Together, these findings establish MSC-EVs as potent cell-free nanotherapeutics that suppress inflammation and provide multiorgan protective effects, offering a novel paradigm for HRS treatment.

## METHODS

### Mice

In this study, C57BL/6 mice (male, 8-10 weeks old), a widely used inbred strain, were purchased from the Laboratory Animal Center of the Fourth Military Medical University, as used in our previous studies^[36,37]^. Animal treatments and the experimental procedures of the present study were performed in accordance with the Guidelines of Intramural Animal Use and Care Committees of The Fourth Military Medical University (approval number: 2020-003) and the Animal Research: Reporting of *In Vivo* Experiments (ARRIVE) guidelines. All mice were housed in the specific pathogen-free housing facility at a constant temperature (21-23 °C) and humidity (45%-50%) in a 12 h light-dark cycle (lights on 07:00-19:00 h), with food and water available *ad libitum* throughout the studies.

The HRS model was established as previously described^[38]^. Briefly, mice were anesthetized, a midline incision was made, and the common bile duct was identified and ligated. In the sham operation, mice underwent laparotomy with bile duct identification and gentle manipulation, but without ligation. Mice were allocated into three groups (6 mice/group) as follows: (1) sham-operated [phosphate-buffered saline (PBS)-treated]; (2) BDL-operated (PBS-treated); (3) BDL + EVs (the MSC-EV dosage was 2 μg/g). One week after BDL, equal volumes of PBS or MSC-EVs were injected intravenously *via* the tail vein as a single dose, and all samples were collected one week later to assess the treatment effect.

### Cell culture

MSCs from the umbilical cord tissues were purchased from the American Type Culture Collection (ATCC, USA, https://www.atcc.org/products/pcs-500-010) and cultured in a humidified atmosphere of 5% CO_2_ at 37 °C. The culture medium consisted of alpha-minimum essential medium (α-MEM, Invitrogen, USA), 10% fetal bovine serum (FBS, Sijiqing, China), 1% penicillin/streptomycin (Invitrogen, USA), and 2 × 10^-3^ M *L*-glutamine (Invitrogen, USA). The cells were passaged with 80%-90% confluence using trypsin (Invitrogen, USA), and the medium was refreshed every 2 days.

### Collection and characterization of MSC-EVs

For MSC-EV collection, as described previously^[39,40]^, the medium was replaced with EV-depleted FBS, obtained by ultracentrifugation at 100,000 × *g* for 18 h, after cells were incubated for 1 day. After 48 h, the supernatant was collected and centrifuged at 800 × *g* for 10 min to remove cells and debris. The supernatant was then further centrifuged at 16,000 × *g* for 30 min and washed twice with PBS to obtain EVs.

For characterization of MSC-EVs, nanoparticle tracking analysis (NTA) was employed to assess the size distribution and was quantified with a PMX Zetaview (Particle Metrix, Germany). The morphology of MSC-EVs was evaluated using transmission electron microscopy (TEM) (Thermo Fisher, USA). The mass spectrometry of MSC-EVs was performed by measuring the total protein concentration using a Bicinchoninic Acid (BCA) Protein Assay Kit (TIANGEN, China).

### Organ distribution of MSC-EVs

For *in vivo* organ distribution, time points were set at 1, 4, 12, and 24 h after MSC-EV administration. MSC-EVs were labeled with 1,1’-dioctadecyl-3,3,3’,3’-tetramethylindotricarbocyanine iodide (DiR; Qiyue Biology, China) and injected intravenously via the caudal vein, and mice were euthanized (*n* = 3). Organs were harvested and imaged using the *In Vivo* Imaging System Lumina XRMS Series 2 (PerkinElmer, USA) to assess MSC-EV biodistribution. Fluorescence intensity was quantified using Living Image software (PerkinElmer, USA).

### Enzyme-linked immunosorbent assay (ELISA)

Serum was isolated from whole peripheral blood extracted from retro-orbital vessels, followed by centrifugation at 1,500 g for 15 min at 4 °C and 12,000 g for another 15 min at 4 °C. The concentration of IL-17 was determined using a commercial kit (Proteintech, China) according to the instructions of the manufacturer.

### Liver and kidney function analysis

To assess the residual function of the injured liver and kidney, mice were euthanized, and serum samples were collected to evaluate liver and kidney function markers, including alanine aminotransferase (ALT) and aspartate aminotransferase (AST) for liver function^[41]^, serum creatinine (Cr) and blood urea nitrogen (BUN) in the assessment of kidney function. Assay kits (Nanjing Jiancheng Bioengineering Institute) were employed to quantify these indices.

### Histological staining

Liver and kidney samples were harvested at the indicated time points, fixed in 4% paraformaldehyde (PFA; Biosharp, China) overnight, and dehydrated through a graded ethanol series. Samples were then cleared with xylene, embedded in paraffin, and sectioned at 5 μm. Hematoxylin and eosin (H&E) and Sirius Red staining were performed using commercial kits (Baso Technology, China).

### Immunofluorescence staining

Liver and kidney tissue samples were fixed in 4% PFA at 4 °C for 4 h. The samples were dehydrated in 30% sucrose for 16-18 h after being washed with PBS. An optimal cutting temperature (OCT) compound (Leica, Germany) was used to embed the samples, and 20 μm cryosections were prepared using a cryostat (CM1950, Leica, Germany) for immunofluorescence staining. Briefly, cryosections were permeabilized with 0.3% Triton X-100 (Sigma-Aldrich, USA) in PBS at room temperature for 20 min. The primary antibodies were incubated with cryosections overnight at 4 °C after blocking in goat serum (Boster, China) for 30 min at room temperature. The following primary antibodies were used: anti-alpha-smooth muscle actin (α-SMA) (Abcam, USA; 1:100), anti-kidney injury molecule-1 (KIM-1) (Abcam, USA; 1:100), anti-phosphorylated mixed lineage kinase domain-like protein (pMLKL) (Bioss, China; 1:100), anti-Lotus tetragonolobus lectin (LTL) (Abcam, USA; 1:100), and anti-IL-17 (Proteintech, China; 1:100). After primary antibody incubation, cryosections were rinsed three times with PBS, and fluorescence-conjugated secondary antibodies were incubated with the cryosections for 1 h at room temperature. The secondary antibodies used were: Cy3 (a cyanine fluorescent dye)-conjugated goat anti-mouse immunoglobulin G (IgG) (YEASEN, China; 1:200), Cy3-conjugated goat anti-rabbit IgG (YEASEN, China; 1:200), and Alexa Fluor 488-conjugated goat anti-mouse IgG (ab150075, Abcam, UK; 1:200). After washing with PBS three times, slides were mounted with Mounting Medium with DAPI (4’,6-diamidino-2-phenylindole)-Aqueous, Fluoroshield (Abcam, UK). Images were acquired using confocal laser scanning microscopy (CLSM) (Nikon A1+, Nikon, Japan) and analyzed using ImageJ software [the National Institute of Health (NIH), USA] by calculating positive area fractions.

### RNA extraction and quantitative real-time polymerase chain reaction analysis

Total RNA was isolated from freshly harvested liver and kidney tissues after homogenization under liquid nitrogen using commercial RNA isolation kits (Foergene, China) according to the manufacturer’s instructions. cDNA was synthesized using a PrimeScript^TM^ RT Reagent Kit (Takara, Japan). Quantitative real-time polymerase chain reaction (qRT-PCR) was then performed using a SYBR Premix Ex Taq II Kit (Takara, Japan) on a Real-Time System (CFX96; Bio-Rad, USA). Quantification was performed using *glyceraldehyde-3-phosphate dehydrogenase* (*GAPDH*) as the internal control, and relative expression levels of each gene were calculated using the 2^-ΔΔCT^ method. The primers used in this study are listed in Supplementary Table 1.

### Proteomic analysis

Protein lysates of MSCs and MSC-EVs were prepared and subjected to liquid chromatography tandem mass spectrometry (LC-MS/MS) analysis on an Orbitrap Exploris 480 mass spectrometer with a NanoSpray III ion source. The Proteome Discoverer system (v2.4.1.15) was used to analyze the raw data, and proteins were identified by comparison against the universal protein resource (UniProt) database, with a false discovery rate (FDR) of 0.01 for both peptides and proteins. Protein quantification was performed using the default parameters in MaxQuant. Functional analysis was conducted based on Gene Ontology (GO) and Kyoto Encyclopedia of Genes and Genomes (KEGG) databases to identify and characterize differentially expressed proteins (DEPs) (fold change > 1.5 and *P*-value < 0.05).

### Statistical analysis

All experiments were performed at least three times independently for each condition to ensure reproducibility. Data are presented as scatter plots with the mean ± standard deviation (SD). Significant differences between groups were determined using two-tailed unpaired Student’s *t*-tests for two-group comparisons and one-way analysis of variance (ANOVA) with Tukey’s post hoc test for multiple-group comparisons. Statistical analyses were performed using GraphPad Prism 8.0.2 software. Differences were considered statistically significant at *P* < 0.05.

## RESULTS

### Liver cholestasis leads to functional impairment of the kidney

**Figure 1:**
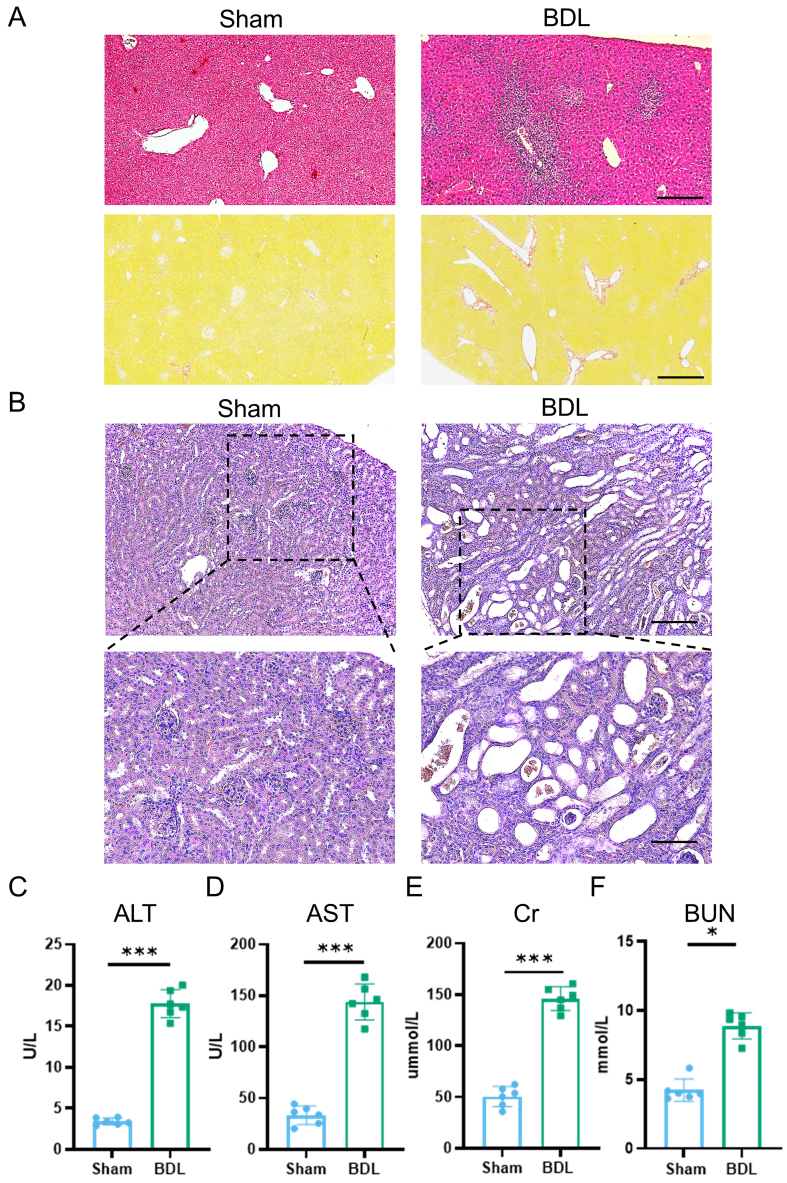
Liver cholestasis leading to functional impairment of the kidney. (A) Histological examination of liver tissue morphology by H&E staining and Sirius Red staining (scale bar = 50 μm); (B) Histological examination of kidney tissue morphology by H&E staining (top image scale bar = 50 μm, bottom image scale bar =100 μm); (C) Serum ALT level in different groups (*n* = 6); (D) Serum AST level in different groups (*n* = 6); (E) Serum Cr level in different groups (*n* = 6); (F) Serum BUN level in different groups (*n* = 6). Data were analyzed by an unpaired two-tailed Student’s *t*-test. Data are presented as mean ± SD. ^*^P < 0.05; ^***^P < 0.001. H&E: Hematoxylin and eosin; ALT: alanine aminotransferase; AST: aspartate transaminase; Cr: creatinine; BUN: blood urea nitrogen; BDL: bile duct ligation.

As previously reported^[42]^, bile cast nephropathy represents a characteristic manifestation of HRS. Accordingly, we established BDL-induced liver fibrosis in mice to model concomitant kidney injury. Histopathological and Sirius Red staining analysis revealed perivascular fibrosis in the liver on day 7 post-BDL [Figure 1A]. Meanwhile, renal H&E staining after BDL showed disordered nephrons, obvious vacuolar degeneration of renal tubular epithelial cells, and dilation of renal tubules containing protein casts [Figure 1B], consistent with phenotypes reported in previous studies^[43,44]^. To further confirm multi-organ damage, serum biomarkers were quantified. BDL mice showed significant elevations in hepatic enzymes, including ALT and AST [Figure 1C and D], and renal dysfunction markers, including Cr and BUN [Figure 1E and F]. These findings validate the BDL model as a robust platform for studying HRS.

### Identification and proteomic profiling of MSC-EVs

**Figure 2:**
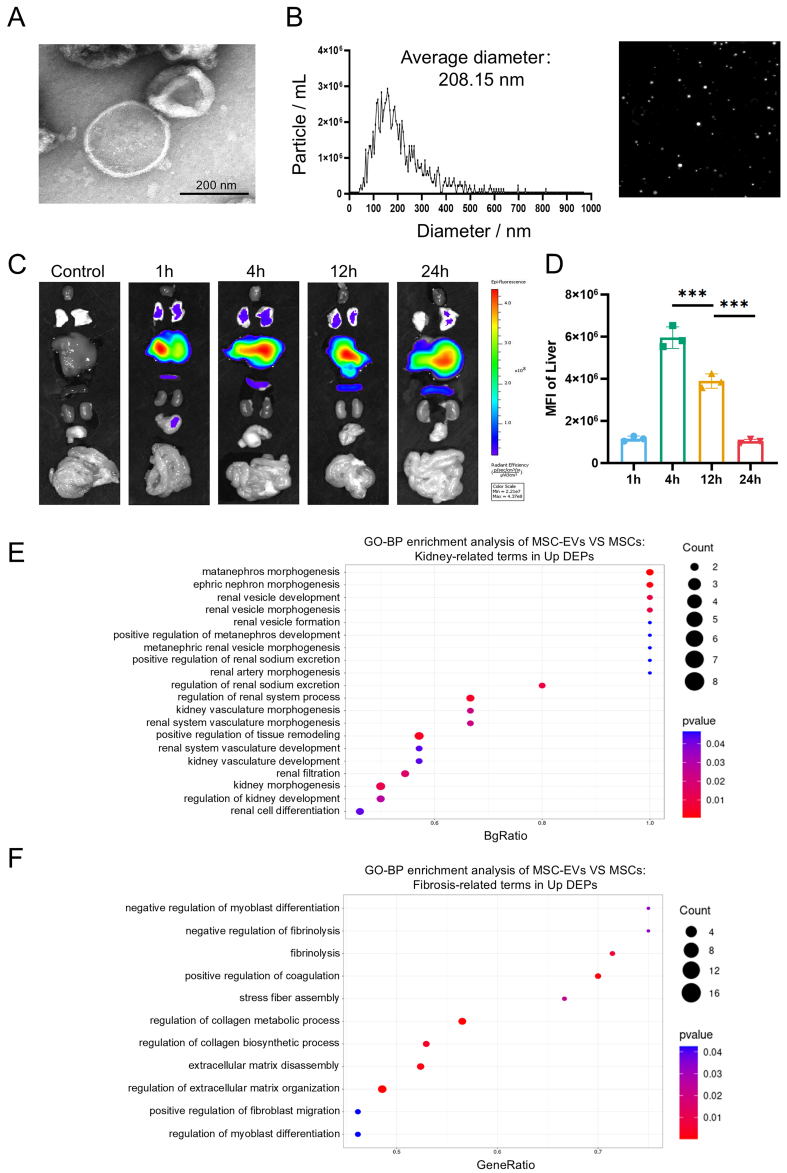
Identification and proteomic profiling of MSC-EVs. (A) Representative TEM image of MSC-EVs (scale bar = 200 nm); (B) Representative NTA image of MSC-EVs; (C) Organ distributions of MSC-EVs in mice detected by Cy5.5 fluorescence after intravenous injection of 30 μg EVs; (D) Statistical analysis of MFI values in the liver (*n* = 3); (E) GO enrichment analysis of significantly upregulated proteins in MSC-EVs. The Y-axis represents kidney-related GO terms, and the X-axis represents BgRatio. The color of the bubble represents enrichment significance and the size of the bubble represents number of upregulated proteins; (F) GO enrichment analysis of significantly upregulated proteins in MSC-EVs. The Y-axis represents fibrosis-related GO terms, and the X-axis represents GeneRatio. Data were analyzed by one-way ANOVA with Tukey’s post hoc test. Data are presented as mean ± SD. ^***^P < 0.001. MSCs: Mesenchymal stem cells; EVs: extracellular vesicles; TEM: transmission electron microscopy; NTA: nanoparticle tracking analysis; Cy5.5: cyanine 5.5; MFI: mean fluorescence intensity; GO: Gene Ontology; ANOVA: analysis of variance; SD: standard deviation.

MSC-EVs confer therapeutic benefits in hepatic or renal pathologies by delivering bioactive macromolecules^[45-48]^. To evaluate their therapeutic potential in HRS, we isolated EVs from umbilical cord mesenchymal stem cells (UCMSCs) *via* differential centrifugation. TEM confirmed characteristic cup-shaped morphology of MSC-EVs [Figure 2A]. Meanwhile, NTA demonstrated a size distribution of 100-200 nm and an average diameter of 208.15 nm [Figure 2B], consistent with typical characteristics of EVs. Upon intravenous infusion, the biodistribution of MSC-EVs showed time-dependent liver uptake, peaking at 4 h [Figure 2C and D]. Next, quantitative proteomics (MSC-EVs *vs*. MSCs) was employed to analyze protein expression, identifying 1,187 proteins that were significantly upregulated in MSC-EVs (Supplementary Figure 1A-C and Supplementary Table 2; FDR < 0.05). To characterize the functional profile of the EV cargo, we performed GO and KEGG enrichment analyses of the upregulated DEPs, and the top 20 terms are shown [Supplementary Figure 2A-D]. Interestingly, a term related to wound healing was significantly enriched in MSC-EVs, suggesting a potential role in tissue repair. To further elucidate the relevance of these upregulated proteins to HRS, we specifically extracted and analyzed GO terms associated with the kidney and anti-fibrosis. The results demonstrated a pronounced association, which included 20 renal-relevant GO entries (*e.g.*, renal vesicle formation, kidney morphogenesis) and 11 enriched GO entries related to anti-fibrosis (*e.g.*, extracellular matrix disassembly, regulation of collagen catabolic process) [Figure 2E and F]. These data indicate that MSC-EVs harbor protein cargos that regulate renal pathophysiology and fibrotic resolution, suggesting an indirect renal-protective potential despite limited renal biodistribution.

### MSC-EVs attenuate histopathological damages of the liver and kidney with restored function

**Figure 3:**
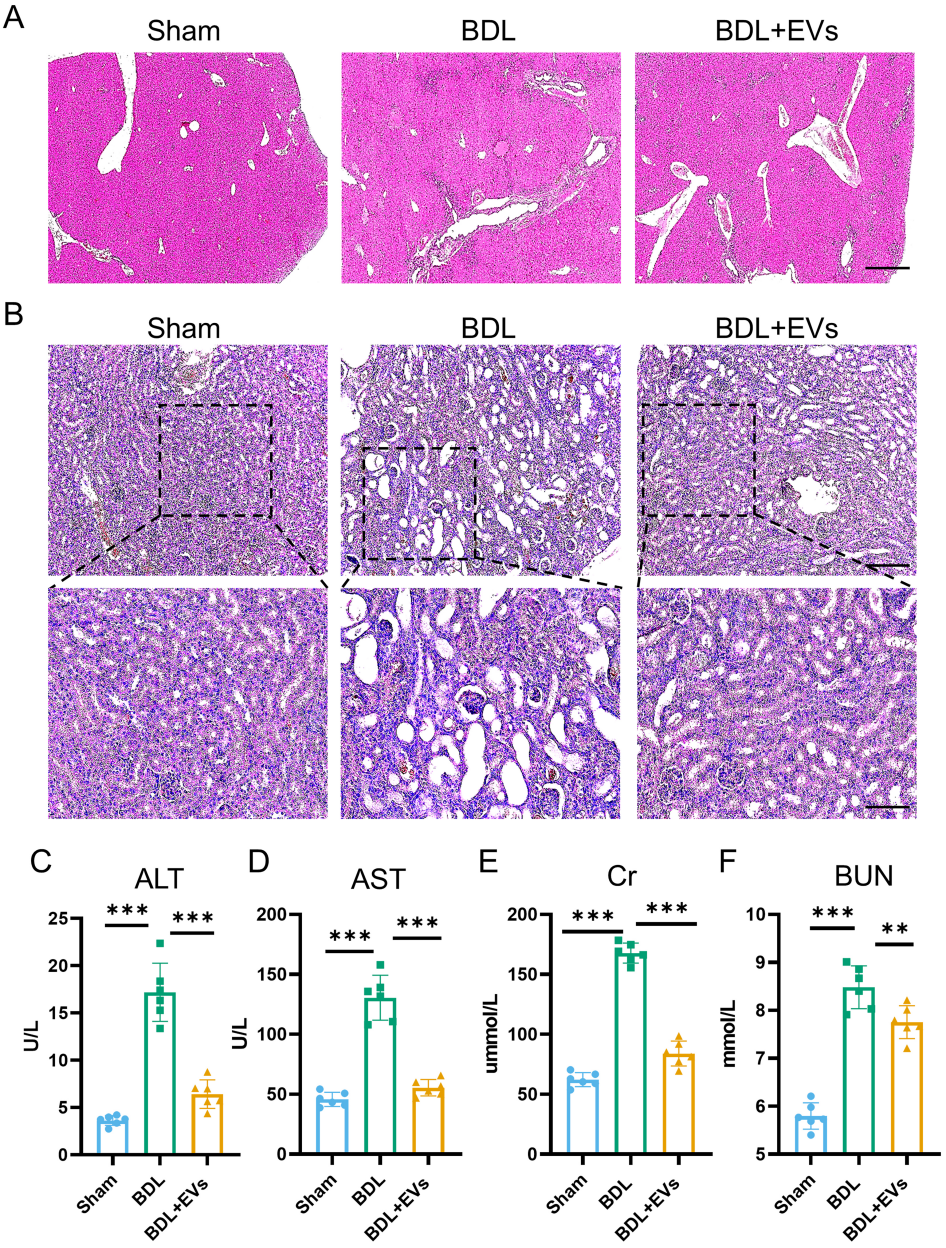
MSC-EVs attenuate histopathological damages and restore the function of the liver and kidney. (A) Histological examination of liver tissue morphology by H&E staining (scale bar = 50 μm); (B) Histological examination of kidney tissue morphology by H&E staining (top image scale bar = 50 μm, bottom image scale bar =100 μm); (C) Serum ALT level in different groups (*n* = 6); (D) Serum AST level in different groups (*n* = 6); (E) Serum Cr level in different groups (*n* = 6); (F) Serum BUN level in different groups (*n* = 6). Data were analyzed by one-way ANOVA with Tukey’s post hoc test. Data are presented as mean ± SD. ^**^P < 0.01; ^***^P < 0.001. MSC-EVs: Mesenchymal stem cell-derived extracellular vesicles; H&E: hematoxylin and eosin; ALT: alanine aminotransferase; AST: aspartate aminotransferase; Cr: creatinine; BUN: blood urea nitrogen; ANOVA: analysis of variance; SD: standard deviation; BDL: bile duct ligation.

Building on proteomic insights into MSC-EV cargos, we next evaluated their therapeutic efficacy in BDL-induced HRS. Mice received a single intravenous MSC-EV injection via the caudal vein at day 7 post-BDL, and tissues and serum were collected 7 days later. Histopathological analysis revealed that MSC-EV treatment significantly mitigated hepatic fibrosis and improved renal architecture compared to the untreated BDL controls [Figure 3A and B, Supplementary Figure 3A and B]. Concordantly, serum biomarkers demonstrated substantial recovery in liver function, with approximately a 50% reduction in ALT and AST [Figure 3C and D]. For kidney function biomarkers, approximately a 39% decrease in Cr and a 20% decrease in BUN were observed [Figure 3E and F]. These findings indicate that MSC-EVs provide marked tissue protection and functional restoration in HRS.

### MSC-EVs ameliorate fibrotic remodeling in both liver and kidney

**Figure 4:**
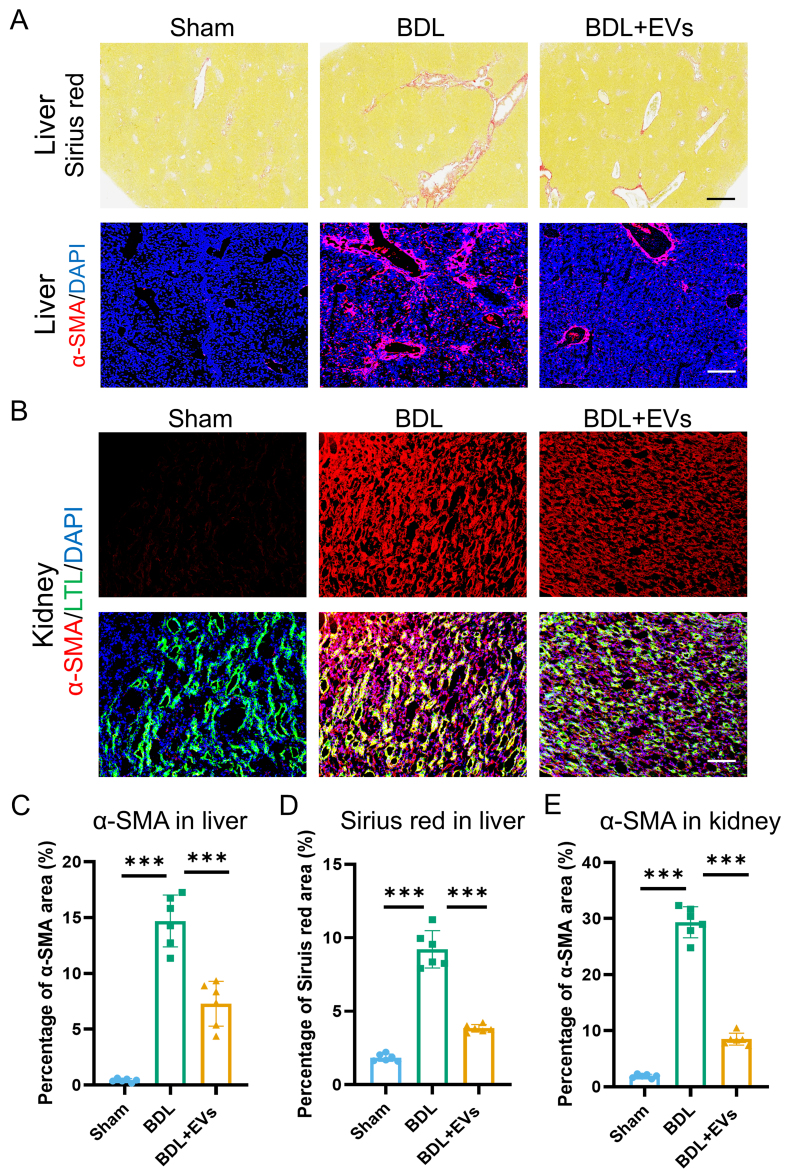
MSC-EVs ameliorate fibrotic remodeling in both liver and kidney. (A) Sirius Red staining and α-SMA (a fibrosis marker) (red)/DAPI (blue) co-immunostaining of Sham, BDL and EV treatment groups in liver (top image scale bar =50 μm, bottom image scale bar =100 μm); (B) Co-immunostaining of LTL (a proximal tubule marker, green), α-SMA (red), and DAPI (blue) in the kidneys of Sham, BDL, and EV-treated groups (scale bar = 100 μm); (C) Quantification of percentage area of Sirius Red in liver (*n* = 6); (D) Quantification of percentage area of α-SMA in the liver (*n* = 6); (E) Quantification of percentage area of α-SMA in the kidney (*n* = 6). Data were analyzed by one-way ANOVA with Tukey’s post hoc test. Data are presented as mean ± SD. ^***^P < 0.001. MSC-EVs: Mesenchymal stem cell-derived extracellular vesicles; α-SMA: alpha-smooth muscle actin; DAPI: 4’,6-diamidino-2-phenylindole; LTL: Lotus tetragonolobus lectin; BDL: bile duct ligation; ANOVA: analysis of variance; SD: standard deviation.

Having established the functional benefits of MSC-EVs, we next assessed their impact on fibrotic pathology in BDL-induced HRS. Sirius Red staining and immunofluorescence of liver sections showed a significant reduction in both collagen deposition and α-SMA expression, a marker of myofibroblast activation, following MSC-EV treatment [Figure 4A and Supplementary Figure 4A and B]. Concomitantly, in renal tissues, immunofluorescence demonstrated that MSC-EV administration substantially attenuated α-SMA expression [Figure 4B]. Quantitative analysis of Sirius Red and α-SMA immunofluorescence staining in liver sections revealed that MSC-EV treatment reduced collagen deposition by 56% and α-SMA-positive area by 47% [Figure 4C and D]. In renal tissues, MSC-EVs attenuated renal interstitial fibrosis, as evidenced by a 72% reduction in α-SMA-positive area [Figure 4E]. To further corroborate the organ-protective effects of MSC-EVs, fibrogenic gene expression was analyzed by qRT-PCR in liver and kidney tissues. MSC-EV administration markedly reduced mRNA levels of collagen, type I, alpha 1 (COL1A1) and α-SMA in BDL-induced HRS, consistent with the observed attenuation of tissue fibrosis [Supplementary Figure 4C-F]. Collectively, these data demonstrate that MSC-EVs elicit potent anti-fibrotic effects in both the liver and kidney, reversing extracellular matrix remodeling in HRS.

### MSC-EVs mitigate hepatic necroptosis and renal tubular injury

**Figure 5:**
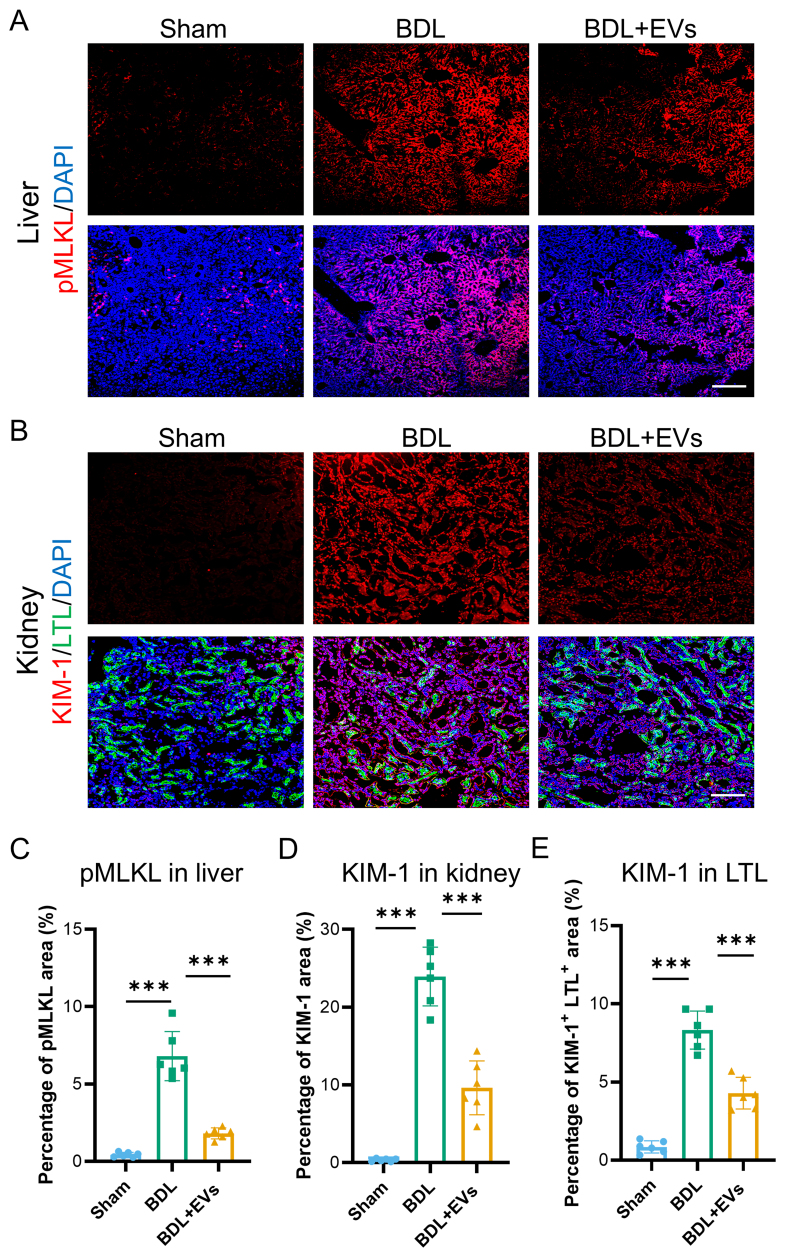
MSC-EVs mitigate hepatic necroptosis and renal tubular injury. (A) pMLKL (the necroptosis marker) (red) and DAPI (blue) co-immunostaining of Sham, BDL and EV treatment groups in liver (scale bar = 100 μm); (B) Co-immunostaining of LTL (a proximal tubule marker, green), KIM-1 (red), and DAPI (blue) in the kidneys of Sham, BDL, and EV-treated groups (scale bar = 100 μm); (C) Quantification of percentage area of pMLKL in liver (*n* = 6); (D) Quantification of percentage area of KIM-1 in the kidney (*n* = 6); (E) Quantification of the percentage area of double-positive KIM-1 and LTL in the kidney (*n* = 6). Data were analyzed by one-way ANOVA with Tukey’s post hoc test. Data are presented as mean ± SD. ^***^P < 0.001. MSC-EVs: Mesenchymal stem cell-derived extracellular vesicles; pMLKL: phosphorylated mixed lineage kinase domain-like; DAPI: 4’,6-diamidino-2-phenylindole; LTL: Lotus tetragonolobus lectin; KIM-1: kidney injury molecule-1; BDL: bile duct ligation; ANOVA: analysis of variance; SD: standard deviation.

Cell death and activation of damage signaling are hallmarks of BDL-induced HRS, in which DAMPs propagate renal injury through systemic inflammation. We then evaluated the potential effects of MSC-EVs on organ-specific damage markers using immunofluorescence. In the liver, MSC-EV treatment profoundly suppressed necroptosis, a key DAMP source, as evidenced by reduced pMLKL expression [Figure 5A and Supplementary Figure 5], a definitive marker of necroptotic execution. Concomitantly, in the kidney, MSC-EVs downregulated KIM-1 expression in the proximal tubular epithelium [Figure 5B]. The therapeutic effects were quantified as a 78% reduction in hepatic pMLKL [Figure 5C], a 65% decrease in whole-kidney KIM-1 area [Figure 5D], and a 45% decrease in tubular KIM-1 intensity [Figure 5E]. These results establish that MSC-EVs concomitantly attenuate hepatic necroptosis and renal tubular damage in HRS.

### MSC-EVs are enriched with immune-regulatory proteins and suppress IL-17-mediated inflammation

**Figure 6:**
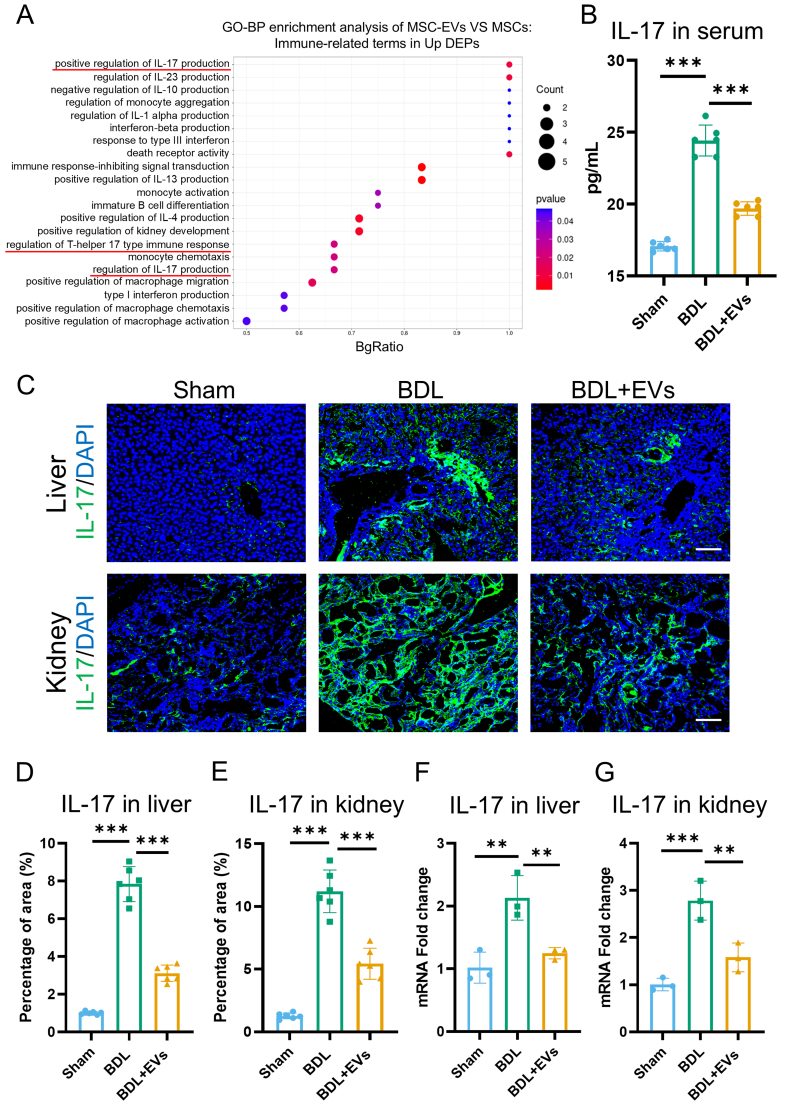
MSC-EVs are enriched with immune-regulatory proteins and attenuate IL-17-mediated inflammation. (A) GO enrichment analysis of significantly upregulated proteins in MSC-EVs. The Y-axis represents immune-related GO terms, and the X-axis represents BgRatio; (B) ELISA analysis of IL-17 in serum (*n* = 6); (C) IL-17 (green) and DAPI (blue) co-immunostaining of Sham, BDL and EV treatment groups in liver and kidney (scale bar = 100 μm); (D) Quantification of percentage area of IL-17 in the liver (*n* = 6); (E) Quantification of percentage area of IL-17 in the kidney (*n* = 6); (F) qRT-PCR analysis of the mRNA expression levels of IL-17 in the liver (*n* = 3); (G) qRT-PCR analysis of the mRNA expression levels of IL-17 in the kidney (*n* = 3). Data were analyzed by one-way ANOVA with Tukey’s post hoc test. Data are presented as mean ± SD. ^**^P < 0.01; ^***^P < 0.001. MSC-EVs: Mesenchymal stem cell-derived extracellular vesicles; GO: Gene Ontology; ELISA: enzyme-linked immunosorbent assay; IL-17: interleukin-17; DAPI: 4’,6-diamidino-2-phenylindole; qRT-PCR: quantitative real-time polymerase chain reaction; mRNA: messenger RNA; BDL: bile duct ligation; ANOVA: analysis of variance; SD: standard deviation.

Systemic inflammation is a pivotal pathogenic factor in HRS that exacerbates renal injury. Beyond terms associated with “wound healing”, GO enrichment analysis of the upregulated DEPs in MSC-EVs revealed significant involvement of immune-related processes, such as “positive regulation of lymphocyte activation” and “regulation of T cell activation” [Supplementary Figure 2A]. Further screening identified 21 GO terms functionally linked to immune regulation within the DEPs, among which IL-17 production was the most enriched [Figure 6A]. To validate these findings functionally, IL-17 expression was quantified in circulation and in target organs. Under the BDL condition, serum IL-17 levels increased, and MSC-EVs were able to reverse this effect [Figure 6B]. Immunofluorescence further demonstrated that MSC-EV treatment significantly suppressed IL-17 expression in both the liver and kidney [Figure 6C-E]. To corroborate this conclusion, qRT-PCR was conducted to assess IL-17 mRNA expression in the liver and kidney, with results consistent with the immunofluorescence data [Figure 6F and G]. These data indicate that MSC-EVs mitigate systemic and local inflammation in the treatment of HRS.

## DISCUSSION

HRS, a critical complication of advanced liver disease, results in > 50% mortality at 3 months and represents a leading indication for combined liver-kidney transplantation^[3]^. This syndrome is characterized by progressive renal dysfunction secondary to systemic hemodynamic and inflammatory dysregulation. Current evidence indicates that portal hypertension-induced splanchnic vasodilation reduces effective circulating volume, triggering compensatory renal vasoconstriction that exceeds physiological adaptation capacity and ultimately diminishes the glomerular filtration rate. While vasoconstrictors and albumin infusion provide symptomatic relief, they fail to address underlying parenchymal damage^[1-3]^. To model this pathophysiology, we established BDL-induced HRS in mice, which recapitulates key clinical features as follows: (1) hepatorenal axis disruption: elevated serum ALT/AST (liver injury) and Cr/BUN (renal dysfunction); (2) histopathological hallmarks: intra-tubular bile casts and tubular dilatation mirroring cholemic nephropathy in humans; and (3) fibrotic progression: perivascular collagen deposition consistent with cholestatic liver disease. This model provides a foundation for subsequent therapeutic investigations.

Stem cell-based therapeutic strategies have demonstrated great potential in preclinical and clinical trials for tissue regeneration and fibrosis treatment. Notably, the advantages of MSCs lie in their ease of extraction and their ability to exert therapeutic effects by homing to damaged areas and regulating the recipient microenvironment^[49-51]^. However, the efficacy of MSC treatment is limited by low cell survival and poor tissue retention^[52-54]^. Crucially, emerging evidence indicates that EVs, with a bilayer lipid membrane structure, can carry signaling biomolecules - including lipids, proteins, and nucleic acids - to mediate intracellular communication under both physiological and pathological conditions^[22]^. Furthermore, EVs play an essential role as mediators of the therapeutic effects of MSC transplantation. Therefore, MSC-EVs, as paracrine products of MSCs, are considered to offer multiple potential advantages as a cell-free alternative to MSC therapy. In addition, MSC-EVs circumvent risks of pulmonary entrapment, tumorigenicity, and immunogenicity while offering scalable production and lyophilization stability^[55]^. In our study, MSC-EVs demonstrated preferential hepatic accumulation. Despite negligible renal uptake, MSC-EV treatment reversed hepatic and renal injuries and restored their function. These findings confirm MSC-EVs as potent cell-free nanotherapeutics for multiorgan recovery in HRS.

Fibrotic remodeling constitutes a pivotal pathological endpoint in HRS, where collagen deposition in both liver and kidney perpetuates organ dysfunction through progressive architectural distortion^[1]^. This process synergizes with inflammation to establish a self-amplifying cycle of organ damage. While MSCs have demonstrated anti-fibrotic potential, their clinical translation is constrained by limited engraftment and transient effects. Importantly, our data establish that MSC-EVs effectively disrupted this fibrogenic cascade, reducing collagen deposition in both liver and kidney. These findings position MSC-EVs as a potent dual-organ anti-fibrotic agent capable of counteracting sustained extracellular matrix remodeling.

The systemic inflammatory response is an important factor that exacerbates the pathological process of HRS. DAMPs and PAMPs are the main molecules that drive systemic inflammation^[56]^. Aggravation of systemic inflammation, in turn, further exacerbates liver and kidney damage. In this study, we demonstrate that MSC-EVs potently disrupted this loop by suppressing hepatic necroptosis and inhibiting renal tubular injury. Beyond targeting upstream DAMPs/PAMPs, our findings reveal that MSC-EVs contribute to the resolution of downstream inflammatory effector responses in HRS. Integrated proteomic and functional analyses demonstrate that MSC-EVs suppressed hepatic IL-17 expression and reduced renal IL-17 intensity by up to 50%, accompanied by ameliorated serum IL-17 levels. These data indicate that MSC-EVs exert immunoregulatory effects in the treatment of HRS.

Our integrated data support a mechanistic model in which the therapeutic effects of MSC-EVs in alleviating HRS are achieved by intercepting the pathogenic crosstalk from the liver to the kidney. The primary site of action is the liver, where MSC-EVs accumulate and inhibit hepatic necroptosis, a key source of pro-inflammatory DAMPs^[57]^. By suppressing this initial injury signal, MSC-EVs prevent the amplification of local and systemic inflammation that drives secondary renal damage. Our proteomic analysis points to the regulation of IL-17-associated pathways as a particularly plausible mediator of this immunomodulatory effect, given the established role of IL-17 in both cholestatic liver injury and renal inflammation^[58,59]^. Further single-cell and spatial transcriptomic analyses will help delineate how EV-induced changes in specific hepatic niches confer remote renal benefits.

Despite promising therapeutic outcomes, this study has several limitations. Firstly, although proteomic analysis identified numerous enriched proteins in MSC-EVs implicated in fibrotic resolution, renal protection, and immune regulation, the specific contributions of individual cargo components to hepatorenal recovery remain unverified. Secondly, although negligible renal EV uptake was observed, significant attenuation of kidney injury occurred, suggesting indirect mechanisms potentially mediated by liver-derived secondary messengers or systemic immunomodulation. The precise effectors remain to be elucidated. Future research should also aim to identify the specific molecular components within MSC-EVs responsible for hepatorenal protection and to optimize these vesicles for clinical applications.

## DECLARATIONS

### Availability of data and materials

The raw sequence data reported in this study have been deposited in the Genome Sequence Archive of the National Genomics Data Center, China National Center for Bioinformation / Beijing Institute of Genomics, Chinese Academy of Sciences (GSA-Human: HRA008022) and are publicly accessible at https://ngdc.cncb.ac.cn/gsa-human. All other data included in this study are available upon reasonable request from the corresponding authors.

### AI and AI-assisted tools statement

Language editing and refinement of selected text passages were performed using AI-assisted tools to improve clarity and conciseness while strictly preserving scientific accuracy. All AI-processed content was verified and approved by the authors. The use of these technologies promotes transparency and trust among authors, readers, reviewers, editors, and contributors, and facilitates compliance with the terms of use of the relevant tools or technologies.

### Copyright

© The Author(s) 2026.
