# Supplementary material for: Multiorgan repair by MSC-derived extracellular vesicles in hepatorenal syndrome through necroptosis alleviation, immune reprogramming and fibrosis resolution
Source: Extracell Vesicles Circ Nucl Acids. 2026 Jan 12;7(1):34–50. doi: 10.20517/evcna.2025.99 (PMC13076343; doi:10.20517/evcna.2025.99)
Supplement: Supplementary file 1 [file evcna-7-1-34-SupplementaryMaterials.pdf]

Supplementary Materials

Multiorgan repair by MSC-derived extracellular vesicles in hepatorenal syndrome through necroptosis alleviation, immune reprogramming and fibrosis resolution

Kai-Chao Zhang[1,2,3], Shi-Han Mu[3,4], Run-Fang Song[2], Yu-Ru Gao[3], Sha Zhang[3,5,6], Chen-Xi Zheng[3], Yan Jin[3], Zhen Gong[7], Bing-Dong Sui[3], Min Zhang[1,2]

[1]College of Life Science, Northwest University, Xi'an 710069, Shaanxi, China.
[2]State Key Laboratory of Military Stomatology & National Clinical Research Center for Oral Disease, Department of General Dentistry and Emergency, School of Stomatology, Fourth Military Medical University, Xi'an 710032, Shaanxi, China.
[3]State Key Laboratory of Oral & Maxillofacial Reconstruction and Regeneration, National Clinical Research Center for Oral Diseases, Shaanxi International Joint Research Center for Oral Diseases, Center for Tissue Engineering, School of Stomatology, Fourth Military Medical University, Xi'an 710032, Shaanxi, China.
[4]Department of Orthodontics, School of Stomatology, The Fourth Military Medical University, Xi'an 710032, Shaanxi, China.
[5]Department of Traditional Chinese Medicine, The First Affiliated Hospital of Fourth Military Medical University, Xi'an 710032, Shaanxi, China.
[6]College of Basic Medicine, Shaanxi Key Laboratory of Research on TCM Physical Constitution and Diseases Prevention and Treatment, Shaanxi University of Chinese Medicine, Xianyang 712046, Shaanxi, China.
[7]Analysis & Testing Laboratory for Life Sciences and Medicine, Fourth Military Medical University, Xi'an 710032, Shaanxi, China.

Correspondence to: Prof. Min Zhang, College of Life Science, Northwest University, Xi'an 710069, Shaanxi, China. E-mail: zhangmin@fmmu.edu.cn; Prof. Bing-Dong Sui, State Key Laboratory of Oral & Maxillofacial Reconstruction and Regeneration, National Clinical Research Center for Oral Diseases, Shaanxi International Joint Research Center for Oral Diseases, Center for Tissue Engineering, School of Stomatology, Fourth Military Medical University, Xi'an 710032, Shaanxi, China.
E-mail: bingdong@fmmu.edu.cn

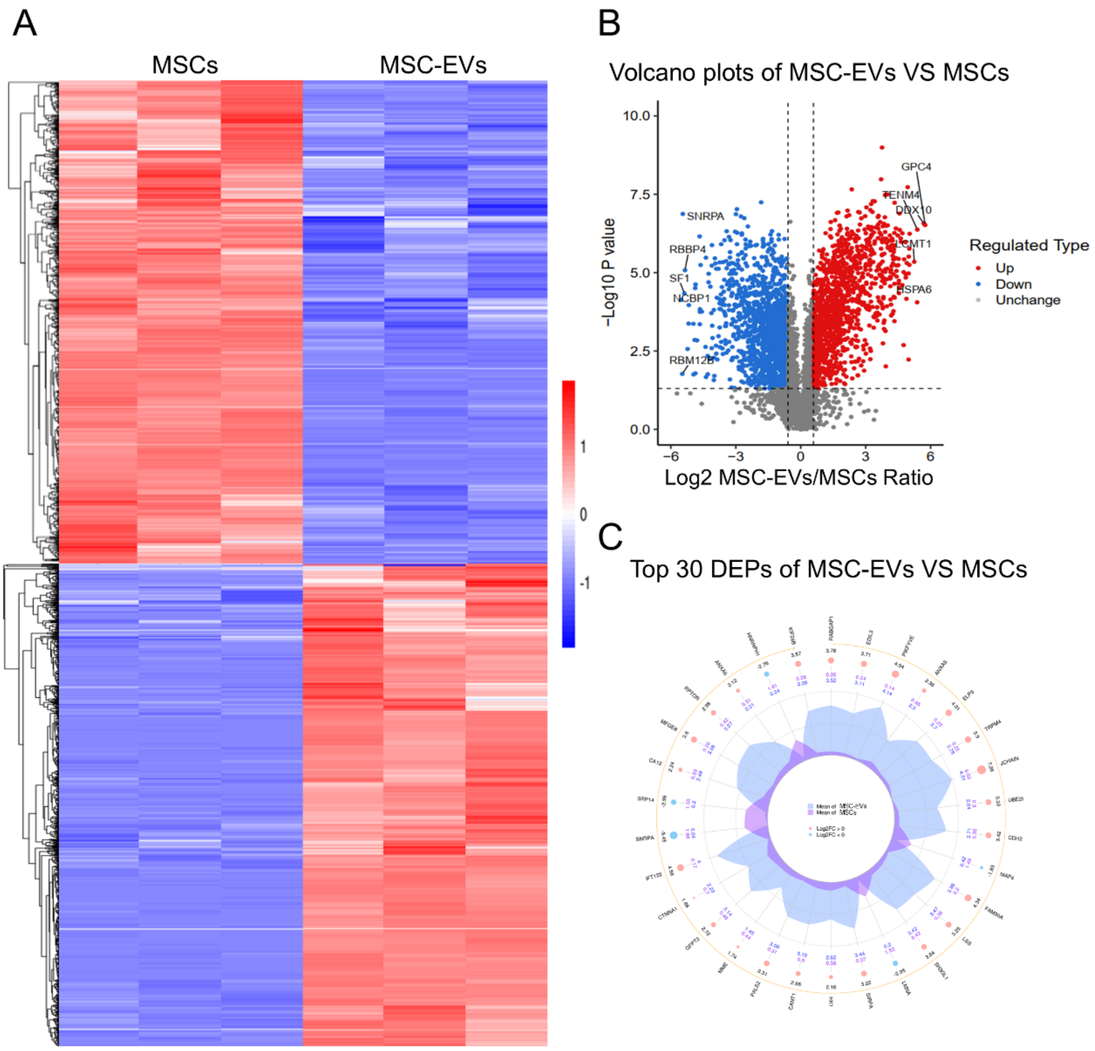

Supplementary Figure 1. Proteomic analysis of MSC-EVs *vs*. MSCs. (A) Heatmap showing significantly upregulated (red dots) and downregulated (blue dots) proteins in MSC-EVs, compared to MSCs; (B) Volcano plot showing significantly upregulated (red dots) and downregulated (blue dots) proteins in MSC-EVs, compared to MSCs; (C) Radar map showing top 30 DEPs of MSC-EVs *vs*. MSCs.

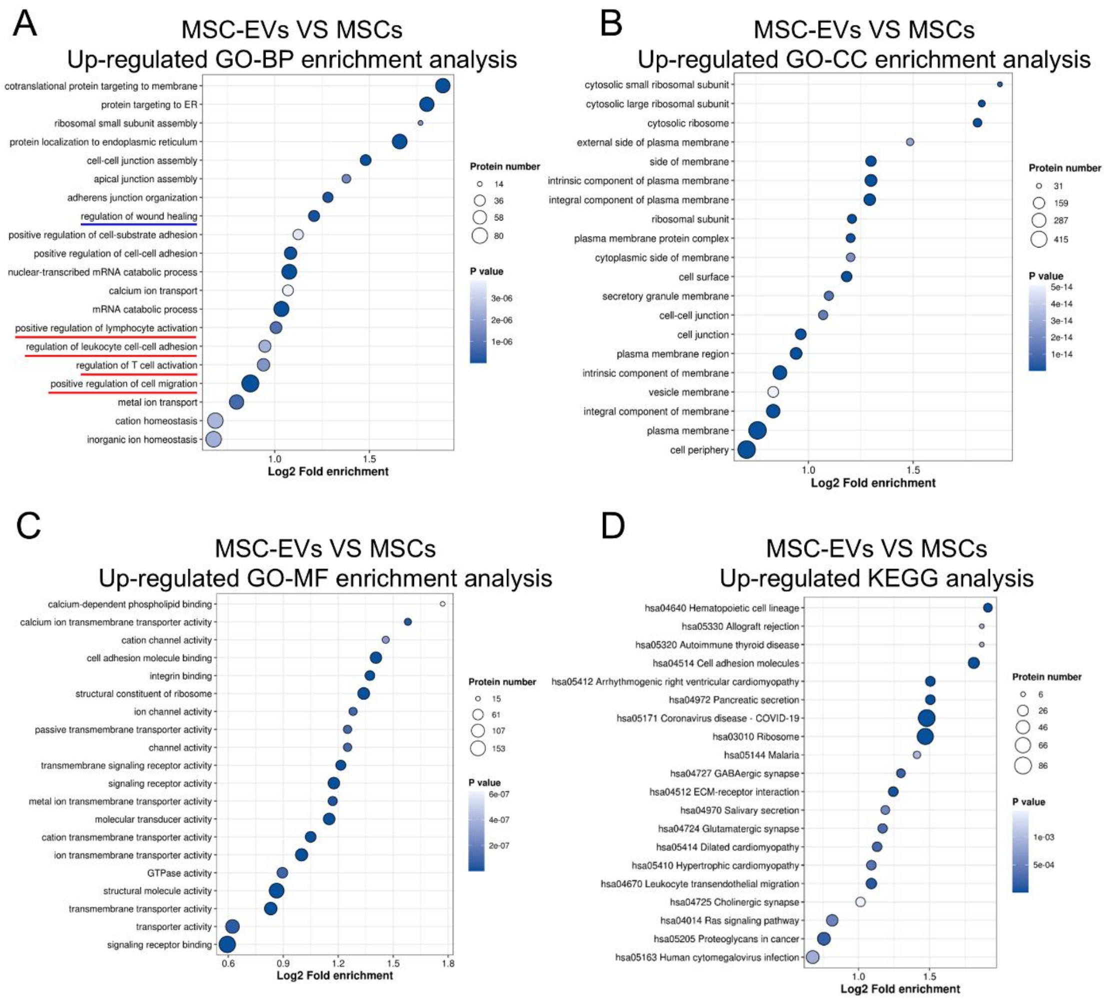

Supplementary Figure 2. Upregulated proteomic enrichment analysis of MSC-EVs *vs*. MSCs. (A) GO-BP enrichment analysis of top 20 up-regulated proteins in MSC-EVs; (B) GO-CC enrichment analysis of top 20 up-regulated proteins in MSC-EVs; (C) GO-MF enrichment analysis of top 20 up-regulated proteins in MSC-EVs; (D) KEGG enrichment analysis of top 20 up-regulated proteins in MSC-EVs.

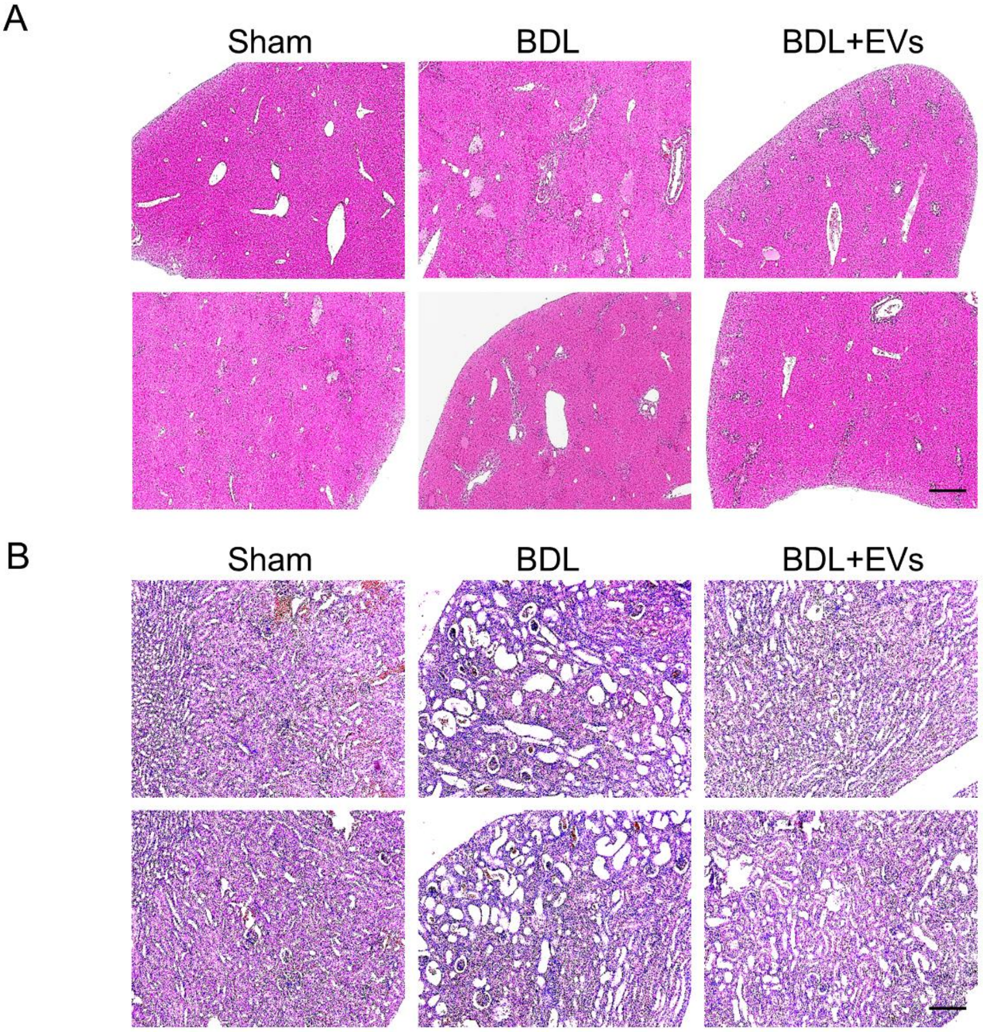

Supplementary Figure 3. MSC-EVs attenuate histopathological damages in liver and kidney. (A) Histological examination of liver tissue morphology by H&E staining (scale bar = 50 μm); (B) Histological examination of kidney tissue morphology by H&E staining (scale bar = 50 μm).

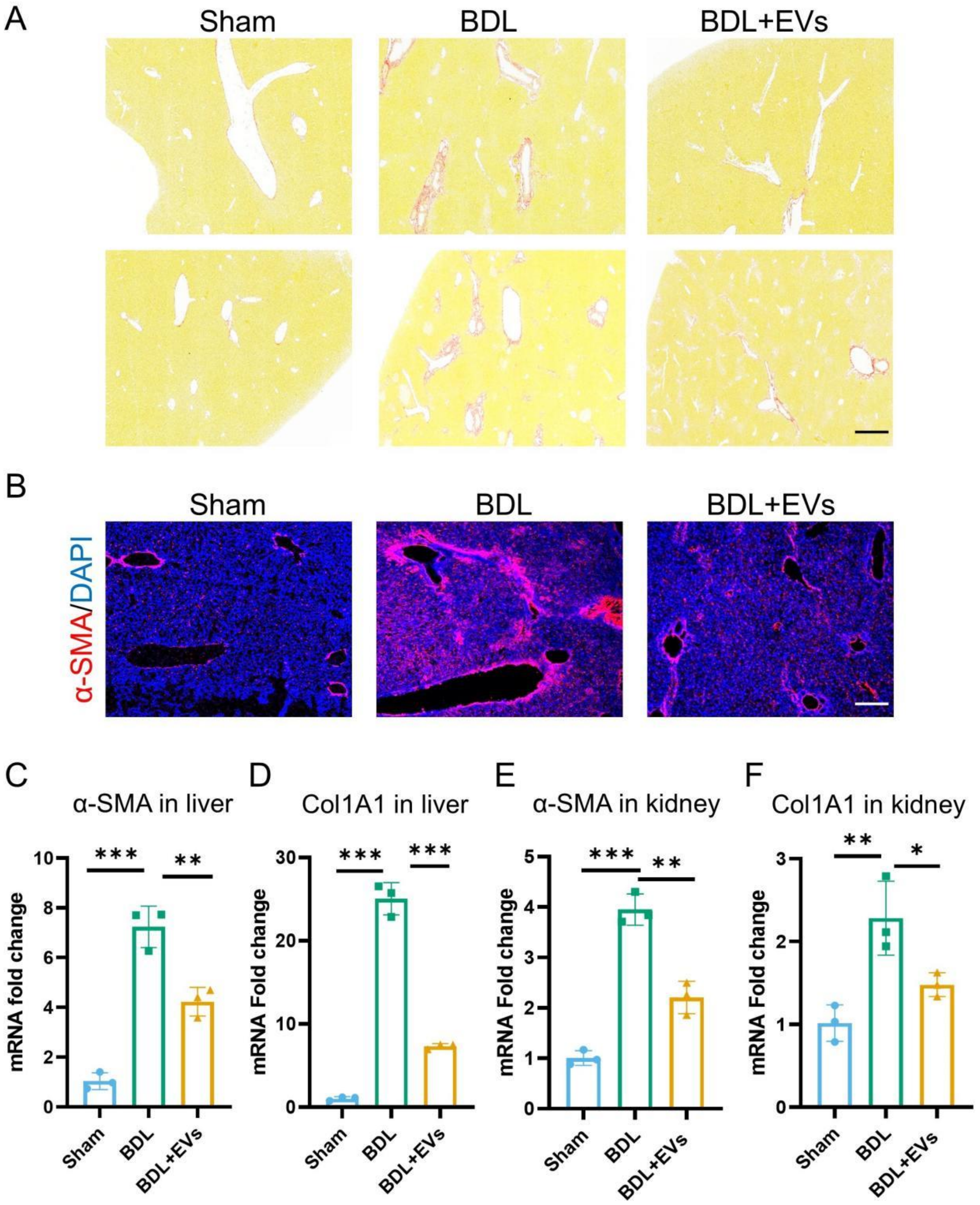

Supplementary Figure 4. MSC-EVs reduce fibrosis in liver and kidney. (A) Histological examination of liver tissue morphology by Sirius red staining (scale bar = 50 μm); (B) α-SMA (red) and DAPI (blue) co-immunostaining of Sham, BDL and EV treatment groups in liver (scale bar =50 μm); (C) qRT-PCR analysis of the mRNA expression levels of *α-SMA* in liver (n = 3); (D) qRT-PCR analysis of the mRNA expression levels of *COL1A1* in liver (n = 3); (E) qRT-PCR analysis of the mRNA expression levels of *α-SMA* in kidney (n = 3); (F) qRT-PCR analysis of the mRNA expression levels of *COL1A1* in kidney (n = 3). Data are presented as mean ± SD. $^{*}P < 0.05$; $^{**}P < 0.01$; $^{***}P < 0.001$.

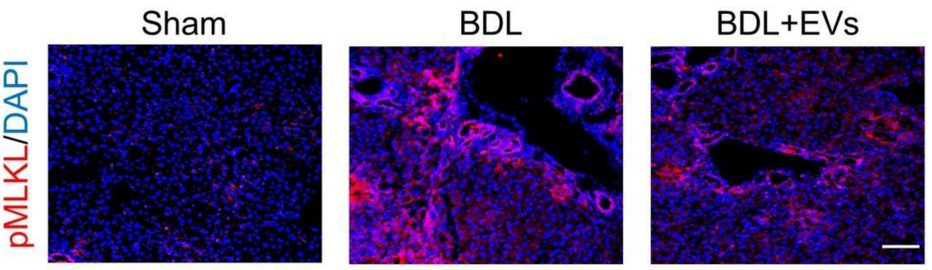

Supplementary Figure 5. MSC-EVs ameliorate liver necroptosis. pMLKL (red) and DAPI (blue) co-immunostaining of Sham, BDL and EV treatment groups in liver (scale bar =100 μm).

Supplementary Table 1. Primers for qRT-PCR

Gene name	Gene primers
GAPDH	F: 5'-TGACCTCAACTACATGGTCTACA-3'
	R: 5'-CTTCCCATTCTCGGCCTTG-3'
IL-17	F: 5'-CTCCAGAAGGCCCTCAGACTAC-3
	R: 5'-GGGTCTTCATTGCGGTGG-3'
α-SMA	F:5'-GTACCACCATGTACCCAGGC-3'
	R:5'-GCTGGAAGGTAGACAGCGAA -3'
COL1A1	F:5'-CGACCTCAAGATGTGCCACT-3'
	R:5'-GCAGTAGACCTTGATGGCGT-3'
